# Supplementary material for: Impact of online lottery sales prohibition on the structure of lottery consumers: A time cost perspective in China
Source: PLoS One. 2025 Dec 5;20(12):e0337433. doi: 10.1371/journal.pone.0337433 (PMC12680344; doi:10.1371/journal.pone.0337433)
Supplement: S1 Appendix — (PDF) [file pone.0337433.s001.pdf]

Supporting information

S1 Appendix. The derivation for value function.

The value function:

$$V(W_t) = max[U(C_t, R_t) + \beta V(W^E_{t+1})] \tag{17}$$

The state variables W_t and W^E_{t+1} is given by:

$$W_t = Y_t(L_t, S_t) + \frac{1+i_{t-1}}{1+\pi_t}(W_{t-1} - C_{t-1}) + J_t$$
$$W^E_{t+1} = Y_{t+1}(L_{t+1}, S_{t+1}) + I^E(W_t - C_t) + J^E_{t+1}(T_t)$$

Substituting the expressions of state variables W_t and W^E_{t+1} into value function (17), we have:

$$\begin{aligned} &V[Y_t(L_t, S_t) + \frac{1+i_{t-1}}{1+\pi_t}(W_{t-1} - C_{t-1}) + J_t] \\ &= maxU\{(C_t, R_t) + \beta V[Y_{t+1}(L_{t+1}, S_{t+1}) + I^E(W_t - C_t) + J^E_{t+1}(T_t)]\} \end{aligned}$$

Subjecting to the wealth constraint and leisure time inequality constraint:

$$W_t = Y_t(L_t, S_t) + \frac{1+i_{t-1}}{1+\pi_t}A_{t-1} + J_t = C_t + A_t$$
$$R^o_t + R^b_t = 1 - L_t - n_t \geq 0$$

The variables that consumers can choose in the current period are $(C^o_t, T^b_t, T^l_t, R^o_t, R^b_t)$. Using the value function to take the derivatives of these decision variables, we obtain the first-order conditions:

$$C^o : U_{C^O_t} = \beta V^{t+1}_w I^E \tag{18}$$
$$T^s : U_{T^b_t} + \beta V^{t+1}_w \sigma^E_s = \beta V^{t+1}_w I^E \tag{19}$$
$$T^w : U_{T^l_t} + \beta V^{t+1}_w \sigma^E_w = \beta V^{t+1}_w I^E \tag{20}$$
$$R^o : U_{R^o_t} = \beta V^{t+1}_w I^E \frac{\partial Y_t}{\partial L_t} \tag{21}$$
$$R^s : U_{T^b_t} \frac{\partial T^b_t}{\partial R^b_t} + U_{R^b_t} + \beta V^{t+1}_w \sigma^E_s \frac{\partial T^b_t}{\partial R^s} = \beta V^{t+1}_w I^E (\frac{\partial Y_t}{\partial L_t} + \frac{\partial T^b_t}{\partial R^b_t}) \tag{22}$$

In the above equation, $U_{C^O_t}$, $U_{T^b_t}$, $U_{T^l_t}$, $U_{R^o_t}$, and $U_{T^b_t}$ are the partial derivatives of the utility function $U(C_t, R_t)$ with respect to C^O_t, T^b_t, T^l_t, R^o_t, and T^b_t, respectively. σ^E_s and σ^E_w are the partial derivatives of $J^E_{t+1}(T_t)$ with respect to T^b_t and T^l_t, respectively. Additionally, $V^{t+1}w$ is a shorthand notation for $V_W(W^E t + 1) = \frac{\partial V(W^E_{t+1})}{\partial W_t}$. Applying the envelope theorem to differentiate the value function with respect to the state variable W_t, we have:

$$W_t : V^t_W = \beta V^{t+1}_w I^E \tag{23}$$

Where V_W^t is the abbreviated notation of $V_W(W_t) = \frac{\partial V(W_t)}{\partial W_t}$, which is the derivative of the value function $V(W_t)$ with respect to the state variable W_t. By applying the Envelope theorem, we can obtain the following equation by combining equations (18), (19), and (20):

$$U_{T_t^b} + \beta V_w^{t+1}\sigma_s^E = U_{T_t^l} + \beta V_w^{t+1}\sigma_w^E = U_{C_t^O} = \beta V_w^{t+1} I^E$$

The economic interpretation of the above equation is that the marginal utility of consuming sports lottery at period t plus the subjective discounted value of the marginal return of sports lottery at period $t+1$ equals the marginal utility of consuming welfare lottery at period t plus the subjective discounted value of the marginal return of welfare lottery at period $t+1$ plus the marginal utility of consuming other goods at time t, all of which are equal to the marginal return of financial assets at period $t+1$. This implies that, given $U_{C_t^O}$, the choice between sports lottery, welfare lottery, and financial assets depends on the consumer's subjective evaluation of the expected returns of these three assets. The asset that is perceived to offer the highest return will be consumed the most.

By combining equations (19), (21), and (22), we obtain:

$$U_{R_t^b} = U_{R_t^o} \tag{24}$$

The economic meaning of equation (24) is that the marginal utility of watching sports events for a consumer at time t is equal to the marginal utility of enjoying other leisure activities. The more leisure time a consumer has, the longer they will spend watching sports programs.

By combining equations (19), (20), and (23), we obtain:

$$U_{T_t^b} = V_W^t(1 - \frac{\sigma_s^E}{I^E})$$
$$U_{T_t^l} = V_W^t(1 - \frac{\sigma_w^E}{I^E})$$

From the above equations, we can recursively derive the marginal utility of lottery consumption for the consumer in period $t+1$:

$$U_{T_{t+1}^b} = V^{t+1}W(1 - \frac{\sigma_s^E}{I^E})$$
$$U_{T_{t+1}^l} = V^{t+1}W(1 - \frac{\sigma_w^E}{I^E})$$

By further combining equation (23), we can obtain the Euler equation for lottery consumption:

$$\frac{U_{T_t^b}}{U_{T_{t+1}^b}} = \frac{U_{T_t^l}}{U_{T_{t+1}^l}} = \beta I^E \tag{25}$$

The economic implication of the above equation is that the greater the subjective expected return on financial assets I^E for the consumer, the greater the marginal utility of lottery consumption U_{T_t} in the current period, and the smaller the quantity of lottery consumption T_t in the current period; or the smaller the marginal utility of lottery consumption $U_{T_{t+1}}$ in the future, and the greater the quantity of lottery consumption T_{t+1} in the future. Similarly, any information that lowers the consumer's subjective expected return on financial assets (such as stock market crashes, economic recessions, or severe inflation) or personal inability to obtain returns from financial

assets will increase the consumer's demand for lottery consumption in the current period.

In order to analyze the effect of purchasing time on consumer's lottery consumption, it is necessary to obtain the relationship between leisure and lottery demand. By combining equations (19), (20), (21), and (23), we can derive the following relationship:

$$U_{T_t^b}\left(\frac{I^E \frac{\partial Y_t}{\partial L_t}}{I^E - \sigma_s^E}\right) = U_{T_t^l}\left(\frac{I^E \frac{\partial Y_t}{\partial L_t}}{I^E - \sigma_w^E}\right) = U_{R_t^o} \tag{26}$$

The above equation can be further simplified to obtain:

$$U_{T_t^b}\left(\frac{\frac{\partial Y_t}{\partial L_t}}{1 - \frac{\sigma_s^E}{I^E}}\right) = U_{T_t^l}\left(\frac{\frac{\partial Y_t}{\partial L_t}}{1 - \frac{\sigma_w^E}{I^E}}\right) = U_{R_t^o} \tag{27}$$
