# Supplementary figures and images for: Impact of online lottery sales prohibition on the structure of lottery consumers: A time cost perspective in China
Source: PLoS One. 2025 Dec 5;20(12):e0337433. doi: 10.1371/journal.pone.0337433 (PMC12680344; doi:10.1371/journal.pone.0337433)

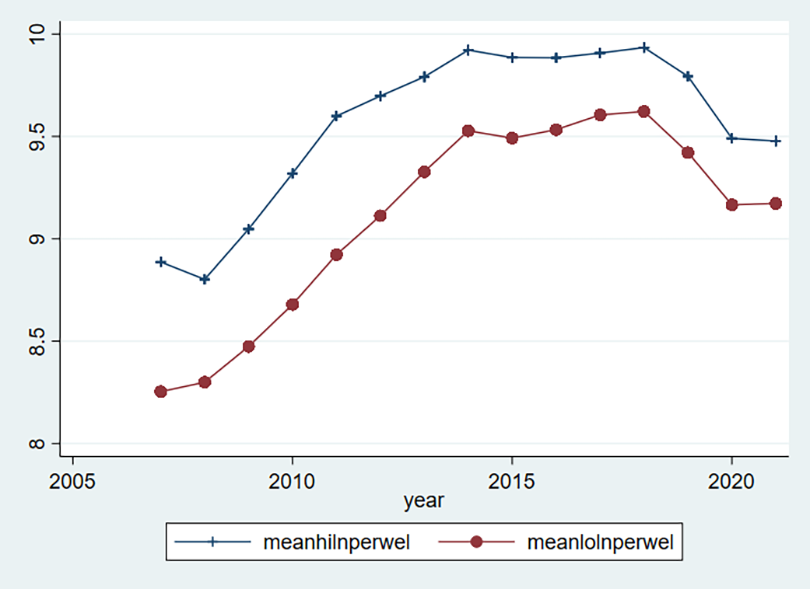

Supplement: S1 Fig — (TIFF) [file pone.0337433.s002.tif]

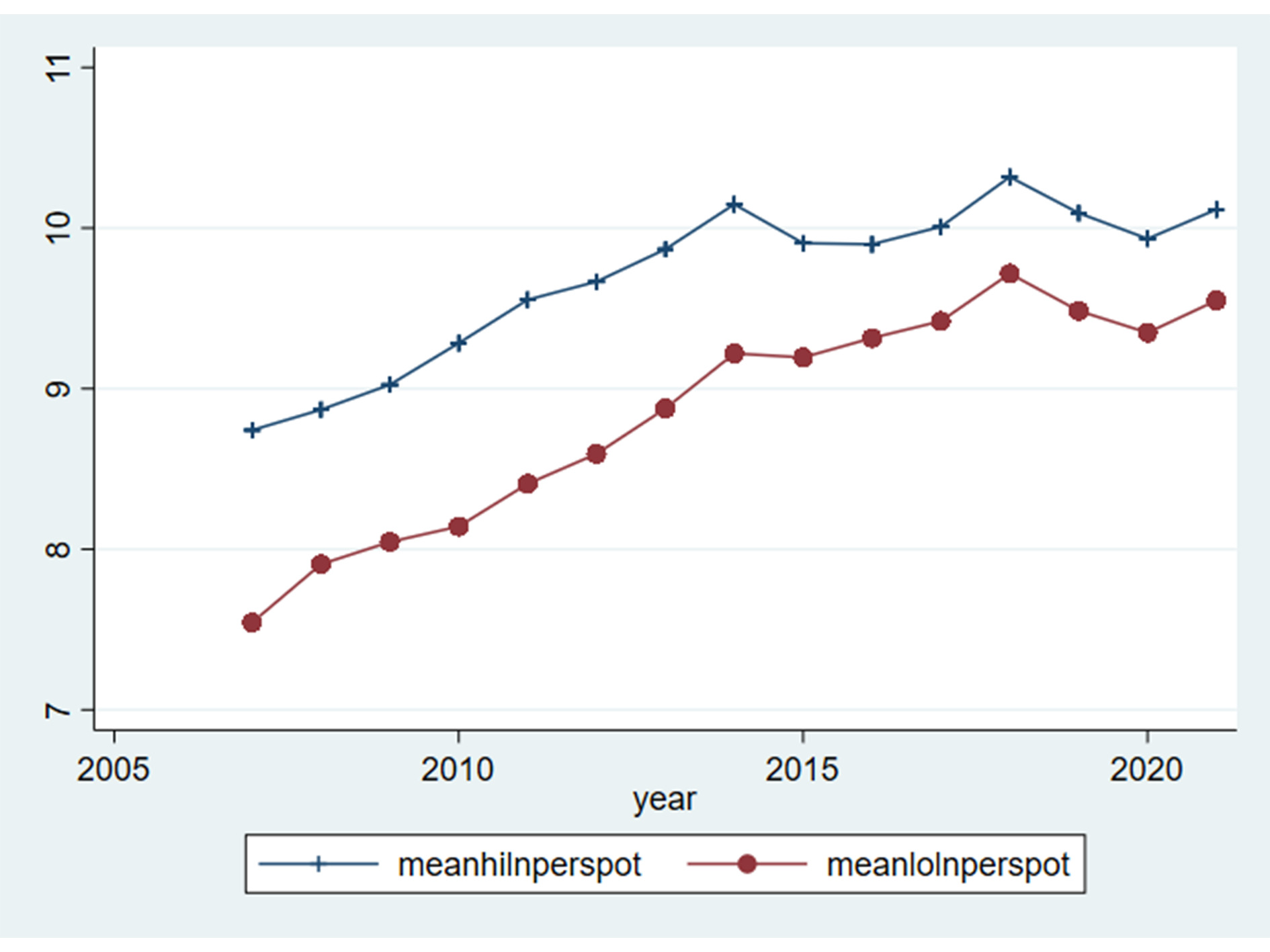

Supplement: S2 Fig — (TIFF) [file pone.0337433.s003.tif]

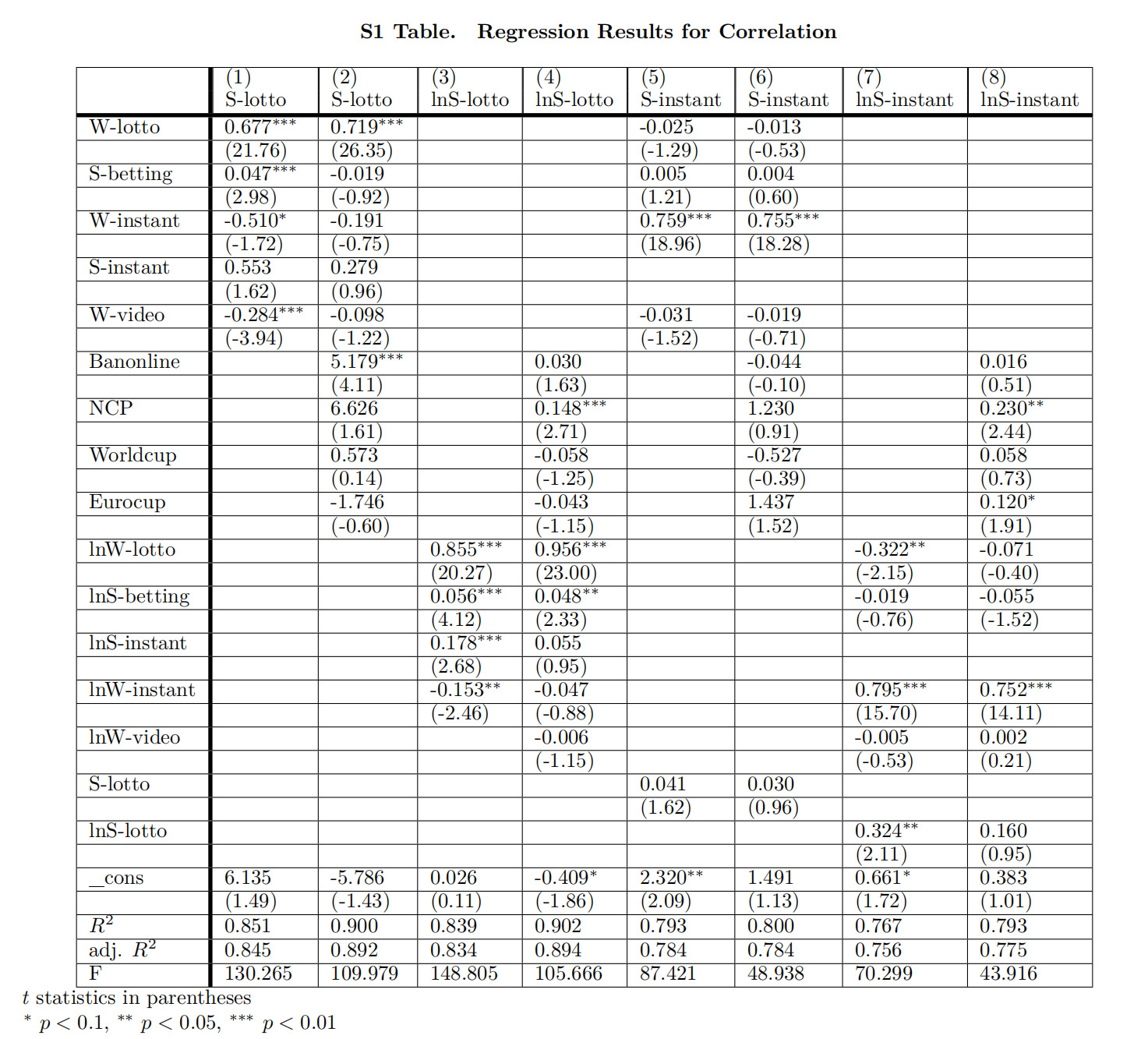

Supplement: S1 Table — (TIFF) [file pone.0337433.s004.tif]

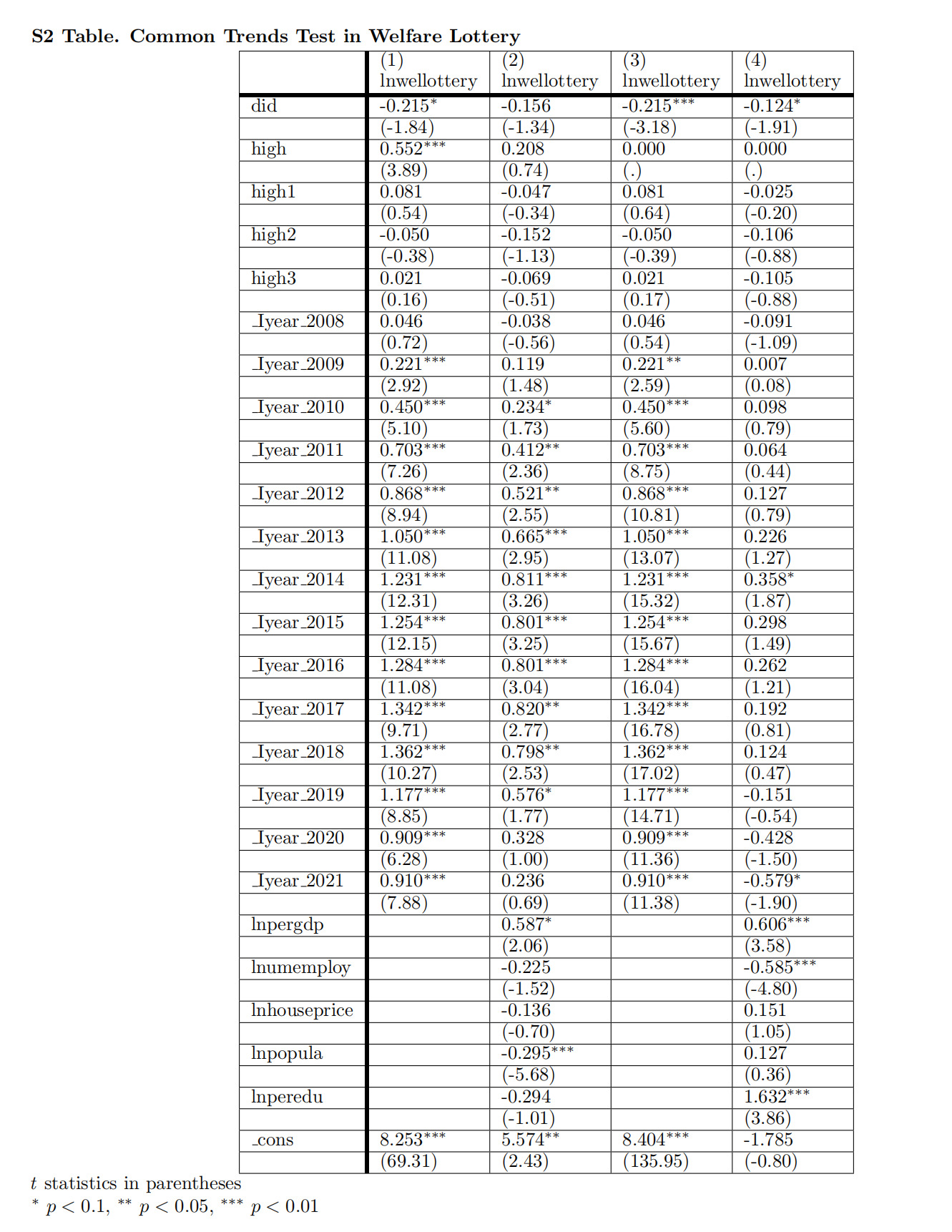

Supplement: S2 Table — (TIFF) [file pone.0337433.s005.tif]

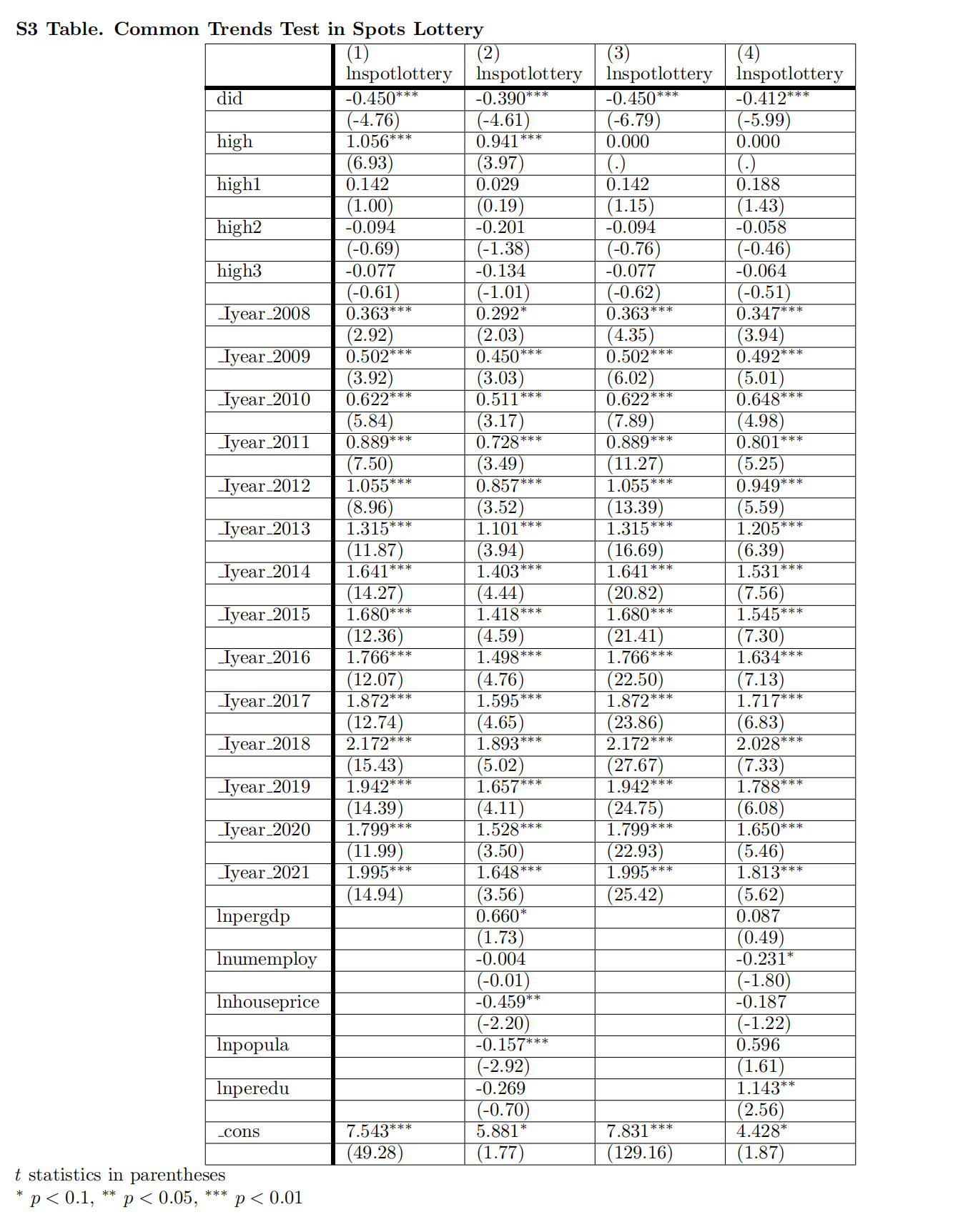

Supplement: S3 Table — (TIFF) [file pone.0337433.s006.tif]
