# Supplementary material for: Impact of online lottery sales prohibition on the structure of lottery consumers: A time cost perspective in China
Source: PLoS One. 2025 Dec 5;20(12):e0337433. doi: 10.1371/journal.pone.0337433 (PMC12680344; doi:10.1371/journal.pone.0337433)
Supplement: Attachment — Submitted filename: Review PONE-D-25-16988.pdf [file pone.0337433.s007.pdf]

Impact of Online Lottery Sales Prohibition on the Structure of Lottery Consumers: A Time Cost Perspective in China

PONE-D-25-16988

The manuscript targets an important aspect of lottery behavior: temporal costs and purchasing convenience. It provides an overview of the evolution of decision theory as it relates to lottery participation — from Von Neumann & Morgenstern's expected utility theory to Gilboa & Schmeidler's MEU, and later behavioral economic theories. In the introduction, the author makes statements that are highly improbable and may be inaccurate. For instance, it is stated that “The earliest research on lotteries traces back to Von Neumann and Mogenstern (1944), who proposed the expected utility theory.” Modern style lotteries have been available as far as the 15$^{th}$ century, in Europe, and became more prevalent in the 18$^{th}$ century. In that sense, it is incorrect to mention that the “earliest research on lotteries traces back” to 1944, as many early scholars have approached the topic albeit in different areas, but the author does not address that.

The study integrates foundational paradoxes (Allais, Ellsberg) with responses from prospect theory and other decision-theoretic innovations. It incorporates non-wealth motivations (Conlisk, Thaler, Nyman), mental accounting, and bounded rationality which illustrates a wide understanding of the intersection between psychology and economic behavior. The mention of the "Stock of Past Gambling" model (Cameron, 2024) shows engagement with very recent theoretical advances.

The manuscript broadens the scope from theoretical models to empirical, demographic-driven research, recognizing heterogeneous motivations across populations. However, the author fails to provide appropriate support on the relevance of the study and the research gap. Additionally, although the review includes literature up to 2024, which is commendable, it would benefit from more engagement with online/mobile lottery behavior, especially given the changing landscape of gambling.

Also worth noting that while the study cites much research, the review lacks critical engagement or evaluation of the limitations of the theories it cites. Are there empirical shortcomings in applying MEU or prospect theory to real-world lottery behavior? How do digital lotteries or gamification challenge classical models?

The text can also benefit from more clarity and an improved writing style. Several phrases are overly complex or awkwardly constructed. As an example: “demonstrating that utility functions with quasi-concave and convex segments could rationalize the welfare-enhancing role of gambling…”. The author should also

be cautious with using repetitive terms, such as “non-wealth motivations,” which appear multiple times without further nuance or variation.

Regarding the Empirical data and strategy: The use of China’s ban on online lottery sales as a natural policy experiment is an interest methodological choice. The claim that online lottery access reduces friction and enhances consumption aligns with broader empirical literature in digital commerce, lending credibility and external validity.

Conceptual Comments: The model is well-grounded in intertemporal utility theory, extending the Money-in-Utility (MIU) framework to include non-monetary consumption, time allocation, and subjective expectations. The choice to integrate non-pecuniary utility and time costs, especially leisure versus labor trade-offs, is highly innovative in the context of lottery consumption. This approach allows you to analyze behavior beyond pure expected monetary gains, addressing the psychosocial appeal of lotteries.

The inclusion of time watching sports ($Rb_t$) as an input into lottery demand is a nuanced feature that reflects real-world engagement with betting-type lotteries. Recognizing that online access dramatically reduces time cost ($n_t \approx 0$) captures a crucial transformation in the gambling industry and is highly policy-relevant.

The paper mentions omitting monetary utility "for simplicity," but this raises significant theoretical concerns. Lotteries are inherently monetary in nature. Ignoring direct monetary utility from consumption limits realism. If monetary gains are excluded from the utility function, why does wealth accumulation ($A_t$, $W_t$) still play a role? The paper could benefit from a clarification whether monetary utility is only excluded in direct utility or if it's integrated implicitly through wealth expectations and constraints.

The model contains numerous simplifying assumptions (constant returns on lottery tickets, fixed subjective expectations, π constant over time, fixed jackpot K). These are useful for tractability, but they limit generalizability. The author could consider explicitly discussing the implications of relaxing some of these (e.g., what happens if $\sigma^E$ varies with K?).

Regarding the link between sports watching and lottery purchases, the assumption is that $\partial Tb_t/\partial Rb_t > 0$ is realistic, but it lacks behavioral micro-foundations. Is this due to increased familiarity? Emotional investment? Perceived competence? The author could provide behavioral or empirical support (e.g., reference dual-process theory, or cite experimental studies on sports betting behavior).

The current OLS framework may suffer from endogeneity, especially considering: Joint marketing strategies; Regional socioeconomic conditions; Time-varying policies (e.g., online sales bans). The paper could consider an instrumental variables (IV) approach or a panel fixed-effects model if time-series or regional data are available.

Causal Interpretation. While the regressions reveal strong correlations, the language used ("explains X% of the variation") verges on causal implication. Suggestion: Include a caution that this is an associational result unless further methods (e.g., DiD, IV) are used to establish causality.

Model (4) posits that a 1% increase in welfare lottery lotto-type sales → 0.956% increase in sports lottery lotto-type sales. I suggest that to expand the discussion on whether this reflects consumer substitution, complementary demand, or co-movement due to third variables (e.g., income spikes or marketing campaigns). Would this relationship hold during economic downturns or shocks?

The text refers to "control variables" but does not specify what these are in the main text. At minimum, list them in a footnote or reference the appendix clearly.

Regarding robustness checks and alternative specifications. Has heteroskedasticity been tested? Were robust standard errors used? Would a Poisson or negative binomial model be more appropriate if sales data are count-based? Briefly mention any such checks performed.

On the 5% Effect of Sports Guessing Games. The text mentions the "impact of sports guessing games is less than 5%," but does not clearly elaborate on why. It should be discussed whether this is due to niche audience segmentation, higher perceived complexity or lower entertainment value; greater time-cost involved in placing sports bets.

The author has hypothesized that high-income regions are more impacted by the online lottery sales ban (especially in sports lottery), but this opens important questions: Are these effects short-term or persistent? Does he observe substitution toward other gambling products (e.g., foreign online platforms or local gaming)? What are the implications for public revenue and responsible gambling policy?
