# Supplementary material for: Impact of online lottery sales prohibition on the structure of lottery consumers: A time cost perspective in China
Source: PLoS One. 2025 Dec 5;20(12):e0337433. doi: 10.1371/journal.pone.0337433 (PMC12680344; doi:10.1371/journal.pone.0337433)
Supplement: Attachment — Submitted filename: Response to Reviewers.pdf [file pone.0337433.s008.pdf]

Response to Reviewers

Reviewer #1：
First, the discussion lacks a balanced interpretation of the results. While the policy may reduce sales, it could ultimately be a net positive for societal health. The lottery in China is intended to serve a welfare purpose, not a wealth distribution one. The author is encouraged to include this perspective in the discussion.

Revision Response:
While prohibiting online lottery purchases has significantly reduced overall lottery sales (particularly for sports lottery's competitive betting products), there exists a structural disparity across different income groups: the decline in purchase volume is more pronounced in high-income regions. From the perspective of social health and the public welfare nature of lotteries in China, the policy's impact presents a "double-edged sword" effect.

On the positive side: By reducing the convenience of purchasing, it may effectively curb some addictive lottery consumption (with a particularly prominent protective effect on low-income groups), which aligns with the core goal of lotteries to serve public welfare and safeguard public well-being.

On the negative side: It has also hindered the widespread participation of high-income groups, whose rational, low-frequency, and small-amount lottery participation is an important force supporting the sustainable growth of public welfare funds.

Therefore, optimizing the convenience of lottery purchases (such as cautiously regulating online channels) is crucial for improving the structure of lottery participants. It can not only expand the participation of high-income groups but also reduce addiction risks through technical controls (e.g., daily betting limits, purchase duration warnings). Ultimately, this will promote the synergy between public welfare fund accumulation and social health development, better fulfilling the welfare mission of lotteries as "taken from the people, used for the people."

Reviewer #1:
Second, the practical implications, including international relevance, are not addressed. For example, as online lottery systems may be relaxed in the future, what recommendations would the author have to strike a balance between sales and addictive behaviors? Additionally, what implications does this have for other countries, especially as online sports lotteries gain popularity worldwide?

Revision Response:
In response to the reviewer's concern regarding the lack of practical implications and international relevance, the following targeted revisions have been made to the conclusion section:

Recommendations for balancing sales and addictive behaviors amid potential future relaxation of online lottery systems: A specific regulatory framework has been added, emphasizing "reliance on an official unified platform for whole-process supervision, including the presetting of daily and weekly betting limits, mandatory display of cumulative purchase duration and consumption amounts, and triggering of automatic reminders or temporary restrictions for high-frequency and high-amount betting behaviors." This measure ensures convenience for ordinary users engaging in low-frequency purchases while directly mitigating addictive consumption risks through technical interventions, addressing the concern of balancing sales growth and addiction prevention.

Supplementary analysis of international relevance: The framework of "official-led platforms integrated with technical rigid constraints" is explicitly highlighted as having international reference value. It provides insights for countries with rapidly popularizing online sports lotteries and underdeveloped regulatory regimes: by incorporating local income stratification characteristics, these countries can prioritize enhancing purchase thresholds through measures such as real-name authentication and account classification to screen for rational participants. Simultaneously, artificial intelligence algorithms can be employed to identify abnormal patterns (e.g., high-frequency betting within short timeframes) for real-time intervention, ultimately facilitating synergy between the sustainable accumulation of public welfare funds and the advancement of social health objectives.

These revisions strengthen the study's practical implications and international relevance through concrete policy recommendations and cross-country adaptability analysis.

Reviewer #1:
Third, this is not a strictly DID analysis, as it lacks a control group. The author should acknowledge this limitation and consider other potential confounders, such as the increased sales during the World Cup in recent years.

Revision Response:
We appreciate Reviewer #1's third comment. Regarding the concern that "this is not a strictly DID analysis due to the lack of a control group," we acknowledge that the design of DID in this study differs from the traditional DID in terms of control group setting, but the core analytical principle remains consistent.

The traditional DID model includes three key variables: a dummy variable distinguishing the period before and after a policy/intervention ($Post_t$ in Figure 1), a dummy variable distinguishing the treatment group from the control group (Treated in Figure 1), and their interaction term (Post×Treated). Its calculation logic is further illustrated in Table 2: within the traditional framework, the differences in outcomes

(ΔY_1, ΔY_0) between the treatment group (i=1) and control group (i=0) before (Period 0, $Post_t=0$) and after (Period 1, $Post_t=1$) the policy implementation are used to calculate the double difference effect ($\Delta\Delta Y=\beta_3$), i.e., the net effect of the policy.

Model:

$$Y_{it} = \alpha + \beta_1 \text{Post}_t + \beta_2 \text{Treated}_i + \beta_3 (\text{Post}_t \times \text{Treated}_i) + \epsilon_{it}$$

where:

- Y_{it} is the outcome variable for entity i at time t.
- Post_t is a dummy variable equal to 1 for periods after the treatment and 0 otherwise.
- Treated_i is a dummy variable equal to 1 for the treatment group and 0 for the control group.
- β_3 is the DiD estimator, representing the treatment effect (ATT).

Fig 1 Traditional DID Analytical Model

Table 2. Interpretation of Parameter Meanings in the Traditional DID Model

Y	Period 0 ($Post_t=0$)	Period 1 ($Post_t=1$)	Difference
Treatment grop(i=1)	$Y_{10}=\alpha+\beta_2$	$Y_{11}=\alpha+\beta_1+\beta_2+\beta_3$	$\Delta Y_1=\beta_1+\beta_3$
Control grop(i=0)	$Y_{00}=\alpha$	$Y_{01}=\alpha+\beta_1$	$\Delta Y_0=\beta_1$
DID			$\Delta\Delta Y=\beta_3$

In our study, the DID model is adaptively adjusted: the "dummy variable distinguishing the treatment group from the control group" is replaced with a "dummy variable distinguishing high-income groups from low-income groups" (as shown in Model 16). However, the core calculation principle of double difference remains unchanged, and the specific logic is detailed in Table 1.

$$T_t^d = \beta_0 + \beta_1 banonline_t + \beta_2 high_i + \beta_3 banonline_t \cdot high_i + \beta_4 control_{it} + \varepsilon_{it} \qquad (16)$$

$$d = \begin{cases} 1, \text{variable is the welfare lottery} \\ 0, \text{variable is the sports lottery} \end{cases} \qquad T_t^d = \begin{cases} T_t^1 = T_{1t}^1 + T_{2t}^1 + T_{3t}^1 \\ T_t^0 = T_{1t}^0 + T_{2t}^0 + T_{3t}^0 \end{cases}$$

Table 1. The meanings of the parameters in Model 16

**Welfare(Sports) lottery**	**Period 0** [1] (Banonline=0)	**Period 1**[2] (Banonline=1)	**Difference**
high-income areas(high)	$\beta_0+\beta_2+\beta_4$	$\beta_0+\beta_1+\beta_2+\beta_3+\beta_4$	$\Delta T_1^d+\Delta T_2^d+\Delta T_3^d=\beta_1+\beta_3$
low-income areas(low)	$\beta_0+\beta_4$	$\beta_0+\beta_1+\beta_4$	$\Delta T_1^d+\Delta T_2^d+\Delta T_3^d=\beta_1$
DID			$\Delta\Delta T_1^d+\Delta\Delta T_2^d+\Delta\Delta T_3^d=\beta_3$

[1]the period before the ban on online sales.
[2]the period after the ban on online sales.

Regarding the "other potential confounders, such as the increased sales during the World Cup in recent years" mentioned by the reviewer, these factors have been incorporated into the model through control variables ($control_{it}$). $control_{it}$ includes a series of exogenous variables that may affect the outcomes, specifically: FIFA World Cup, COVID-19 pandemic, benchmark interest rate, population, per capita GDP, per capita education, and housing prices.

Reviewer #2:
In the introduction, the author makes statements that are highly improbable and may be inaccurate. For instance, it is stated that “The earliest research on lotteries traces back to Von Neumann and Mogenstern (1944), who proposed the expected utility theory.” Modern style lotteries have been available as far as the 15th century, in Europe, and became more prevalent in the 18th century. In that sense, it is incorrect to mention that the “earliest research on lotteries traces back” to 1944, as many early scholars have approached the topic albeit in different areas, but the author does not address that.

Revision Response:
We appreciate Reviewer #2’s insightful comment on the inaccuracy in the introduction. Regarding the statement that "the earliest research on lotteries traces back to Von Neumann and Mogenstern (1944), who proposed the expected utility theory," we acknowledge its imprecision and would like to clarify and revise it.

As noted by the reviewer, modern-style lotteries emerged as early as the 15th century in Europe and became more prevalent in the 18th century. Our original wording failed to distinguish between "the history of lottery practice" and "academic research on lotteries," leading to ambiguity. In fact, what we intended to emphasize is that within the framework of modern economic theory, Von Neumann and Mogenstern’s (1944) expected utility theory laid a foundational groundwork for systematic academic research on lottery-related behaviors. This theory provided core analytical tools for subsequent studies on risk preference, utility evaluation, and decision-making in lottery participation.

To address the oversight of early scholars’ discussions on lottery-related topics in other fields, we will supplement relevant historical context in the revised manuscript, clearly differentiating between the long history of lottery practice and the starting point of modern academic research on this subject to avoid misunderstanding.

Reviewer #2:
The manuscript broadens the scope from theoretical models to empirical, demographic-driven research, recognizing heterogeneous motivations across populations. However, the author fails to provide appropriate support on the relevance of the study and the research gap. Additionally, although the review includes literature up to 2024, which is commendable, it would benefit from more engagement with online/mobile lottery behavior, especially given the changing landscape of gambling.

Revision Response:
We sincerely appreciate Reviewer #2’s valuable feedback, which has helped strengthen the manuscript. We have addressed the concerns raised by enhancing the relevance of the study, clarifying research gaps, and deepening engagement with online/mobile lottery behavior literature, as detailed below:

1. Strengthening Support for Study Relevance and Research Gaps

To address the need for clearer support on relevance and research gaps, we have revised the literature review to explicitly identify critical limitations in existing scholarship within the context of digitalized gambling:

Under-exploration of purchasing convenience across demographics and lottery types: While prior research acknowledges purchasing convenience as a factor in consumption, it rarely examines how this factor shapes lottery behavior differentially across income groups or lottery categories (e.g., fixed-prize vs. progressive jackpots). This is a key gap, as offline studies have already documented income-based disparities in lottery participation, but their digital extensions remain underexplored.

Mismatch between traditional theories and digital contexts: Classical theoretical frameworks, developed for offline settings, struggle to explain the unique dynamics of digital lottery environments—such as real-time accessibility and mobile interactions—that reshape consumption decisions.

Overemphasis on sports betting in online gambling research: Existing online gambling studies focus heavily on sports betting, which differs from lotteries in core motives (skill vs. luck) and audience structure, leaving critical gaps in understanding online lottery-specific mechanisms.
These gaps underscore the relevance of our study: by focusing on temporal costs and leveraging China’s unique policy context of "prohibiting online lottery sales" as a natural instrument, we address these limitations to provide new insights into digital lottery behavior.

2. Enhancing Engagement with Online/Mobile Lottery Behavior Literature

We have significantly expanded engagement with online/mobile lottery behavior research, integrating key findings that reflect the changing gambling landscape:

Psychological and structural mechanisms: Early studies highlight how mobile platforms, enabled by smartphone accessibility, interact with associative learning to accelerate maladaptive behaviors, while digitized lottery markets drive addiction through omnilocal accessibility and transactional immediacy—factors that eliminate physical constraints, truncate deliberation intervals, and amplify impulsivity (James et al., 2017; Churchill and Farrell, 2018; Zhang et al., 2022).

Behavioral drivers and impacts: Subsequent research clarifies that real-time excitement, smartphone-enabled features (e.g., "cash-out"), and targeted marketing shape online gambling engagement, particularly among young adults, with promotional tactics reducing perceived risk and fueling impulsive behavior (Killick

and Griffiths, 2021; Dunlop and Ballantyne, 2021; Killick and Griffiths, 2022; Reeve and Pincin, 2025).

Digital efficiency and expenditure: Recent work underscores that instantaneous, ubiquitous access to betting apps—coupled with platform functionality and social influences—facilitates harmful behaviors (e.g., increased frequency, impulsive wagering) among young adults, while U.S. states with high online lottery penetration demonstrated faster post-pandemic revenue recovery, confirming temporal efficiency as a critical driver of amplified spending (Hing et al., 2024; Hickman, 2025).

These additions ensure the review comprehensively reflects the multifaceted impact of digitalization on gambling behavior, aligned with the evolving landscape of online/mobile lottery.
We believe these revisions effectively address the reviewer's concerns, enhancing the manuscript's rigor and relevance. Thank you again for your constructive input.

Reviewer #2:
Also worth noting that while the study cites much research, the review lacks critical engagement or evaluation of the limitations of the theories it cites. Are there empirical shortcomings in applying MEU or prospect theory to real-world lottery behavior? How do digital lotteries or gamification challenge classical models?

Revision Response:
We appreciate Reviewer #2's insightful observation regarding the need for critical engagement with the theoretical limitations cited in the manuscript. To address this, we have incorporated an explicit evaluation of the empirical shortcomings of prospect theory and Maxmin Expected Utility (MEU) theory in explaining real-world lottery behavior, particularly in digital contexts.

Specifically, we note that prospect theory, with its focus on static decision-making and challenges in accounting for individual parameter variability, fails to capture dynamic adjustments like loss chasing and social influences on lottery participation. Meanwhile, MEU theory's assumptions of perfect rationality and fixed risk attitudes cannot explain the overestimation of small probabilities, entertainment-driven motives, or context-dependent risk preferences inherent in lottery behavior. These limitations are further amplified by digital lotteries, where gamified features like instant feedback distort risk perceptions in ways that classical models cannot encompass.

This critical engagement with theoretical limitations strengthens the manuscript's rigor and contextualizes the need for our study, which leverages a temporal cost framework to address these gaps in understanding digital lottery behavior. Thank you for highlighting this important aspect.

Reviewer #2:

The text can also benefit from more clarity and an improved writing style. Several phrases are overly complex or awkwardly constructed. As an example: “demonstrating that utility functions with quasi-concave and convex segments could rationalize the welfare-enhancing role of gambling...”. The author should also be cautious with using repetitive terms, such as “non-wealth motivations,” which appear multiple times without further nuance or variation.

Revision Response:
We sincerely appreciate Reviewer #2’s feedback on enhancing clarity, refining writing style, and addressing repetitive terminology. To address these concerns, we have made the following revisions:

Removing overly complex phrasing: The sentence referencing Hartley and Farrell (2002), which was noted as awkwardly constructed, has been removed from the manuscript. This streamlines the narrative without compromising the core theoretical framework.

Adding nuance to repetitive terms: For the term “non-wealth motivations,” we have added a concise explanation after its first appearance: “i.e., non-monetary benefits such as entertainment, social interaction, or the psychological thrill of anticipation”. This clarifies the concept and reduces redundancy in its subsequent mention, providing greater nuance to the discussion.

These revisions aim to improve readability and precision, ensuring the literature review is more accessible while maintaining academic rigor. Thank you for your valuable suggestions.

Reviewer #2:
The paper mentions omitting monetary utility "for simplicity," but this raises significant theoretical concerns. Lotteries are inherently monetary in nature. Ignoring direct monetary utility from consumption limits realism. If monetary gains are excluded from the utility function, why does wealth accumulation (A_t, W_t) still play a role? The paper could benefit from a clarification whether monetary utility is only excluded in direct utility or if it's integrated implicitly through wealth expectations and constraints.

Revision Response:
We thank Reviewer #2 for highlighting the need to clarify the treatment of monetary utility in our model, a critical point for theoretical consistency. To address this, we have revised the relevant statement to explicitly distinguish between excluded and retained components of monetary utility:
“To simplify the model, we exclude non-wealth monetary utility—such as transactional convenience or subjective satisfaction from holding cash—while retaining the wealth utility of money, which remains central to the analysis.”

This revision clarifies that our simplification omits only non-wealth dimensions of monetary utility (e.g., psychological or transactional benefits of holding cash) rather than all monetary considerations. The wealth utility of money—reflected in variables like asset accumulation (A_t) and total wealth (W_t)—remains integral to the framework, aligning with the inherently monetary nature of lotteries. This distinction resolves the theoretical concern by explicitly linking retained wealth utility to the role of A_t and W_t in the model, ensuring realism while maintaining analytical tractability.

We appreciate this feedback, which has strengthened the precision of our theoretical exposition.

Reviewer #2:
The model contains numerous simplifying assumptions (constant returns on lottery tickets, fixed subjective expectations, π constant over time, fixed jackpot K). These are useful for tractability, but they limit generalizability. The author could consider explicitly discussing the implications of relaxing some of these (e.g., what happens if σ^E varies with K?).

Revision Response:
We appreciate Reviewer #2's thoughtful comments on the model's simplifying assumptions and their implications for generalizability. We acknowledge that these assumptions, while aiding analytical tractability, have limitations, and we clarify their rationale and potential extensions as follows:
First, regarding the fixed jackpot K and constant returns on lottery tickets (σ_E): In China's institutional context, welfare and sports lotteries have maintained explicit prize caps for over a decade, with minimal adjustments to jackpot structures. This stability justifies treating K as fixed and σ_E (subjective expected marginal returns) as constant in the current analysis, which focuses on the impact of lottery purchasing convenience (e.g., online sales prohibition) rather than jackpot dynamics. That said, we recognize that relaxing this assumption—for instance, allowing σ_E to vary with K—would be valuable. Our subsequent research is already exploring this direction, particularly how σ_E responds non-linearly to K, with explosive growth occurring once K crosses a certain threshold, as observed in markets with flexible prize structures.

Second, the assumption of fixed subjective expectations and constant inflation (π) reflects practical constraints: Subjective expectations are inherently difficult to quantify with existing data, and inflation's multifaceted, indirect effects on lottery behavior would complicate isolating the core mechanism of interest (temporal cost impacts). Relaxing these assumptions could reveal richer dynamics—for example, how shifting expectations or inflation volatility interact with purchasing convenience—but would require more granular data on individual beliefs and macroeconomic shocks, which are beyond the scope of the current study.

These clarifications highlight that while the model's simplifications are grounded in institutional realities and analytical focus, relaxing them (as planned in follow-up work) could enhance generalizability. We thank the reviewer for this insight, which underscores avenues for future research.

Reviewer #2:
Regarding the link between sports watching and lottery purchases, the assumption is that $\partial Tb_t/\partial Rb_t > 0$ is realistic, but it lacks behavioral micro-foundations. Is this due to increased familiarity? Emotional investment? Perceived competence? The author could provide behavioral or empirical support (e.g., reference dual-process theory, or cite experimental studies on sports betting behavior).

Revision Response:
We appreciate Reviewer #2's valuable feedback on strengthening the behavioral micro-foundations for the assumption $\partial Tb_t/\partial Rb_t > 0$. To address this, we have supplemented empirical and theoretical support as follows:

We now explicitly cite empirical evidence confirming the positive correlation between sports viewing and lottery purchases: Yao et al. (2024) demonstrate that increased viewing duration during major events like the World Cup directly stimulates lottery purchases, while Lin et al. (2022) highlight that spectators' engagement—including emotional investment and interest—enhances consumption motives to drive lottery decisions.

To clarify the behavioral mechanisms, we incorporate dual-process theory (Kahneman, 2011), explaining that sports viewing activates "fast thinking," which lowers rational evaluation thresholds and facilitates emotionally driven lottery participation. This connects the observed empirical patterns to underlying psychological processes, addressing concerns about micro-foundations.

These revisions strengthen the theoretical and empirical grounding of the assumption, ensuring clearer support for the link between sports viewing and lottery purchases. Thank you for this insightful suggestion.

Reviewer #2:
The current OLS framework may suffer from endogeneity, especially considering: Joint marketing strategies; Regional socioeconomic conditions; Time-varying policies (e.g., online sales bans). The paper could consider an instrumental variables (IV) approach or a panel fixed-effects model if time-series or regional data
are available.
Causal Interpretation. While the regressions reveal strong correlations, the language used ("explains X% of the variation") verges on causal implication. Suggestion: Include a caution that this is an associational result unless further methods (e.g., DiD, IV) are used to establish causality.

Model (4) posits that a 1% increase in welfare lottery lotto-type sales → 0.956% increase in sports lottery lotto-type sales. I suggest that to expand the discussion on whether this reflects consumer substitution, complementary demand, or co movement due to third variables (e.g., income spikes or marketing campaigns).Would this relationship hold during economic downturns or shocks?
The text refers to "control variables" but does not specify what these are in the main text. At minimum, list them in a footnote or reference the appendix clearly.

Regarding robustness checks and alternative specifications. Has heteroskedasticity been tested? Were robust standard errors used? Would a Poisson or negative binomial model be more appropriate if sales data are count based? Briefly mention any such checks performed.

On the 5% Effect of Sports Guessing Games. The text mentions the "impact of sports guessing games is less than 5%," but does not clearly elaborate on why. It should be discussed whether this is due to niche audience segmentation, higher perceived complexity or lower entertainment value; greater time-cost involved in placing sports bets.

Revision Response:
We sincerely appreciate Reviewer #2's insightful comments on endogeneity, causal interpretation, and model specifications, which have guided important revisions to enhance the manuscript's rigor. We address each concern as follows:

1. Endogeneity and OLS Framework Rationale
We acknowledge that the OLS framework may be susceptible to endogeneity from factors like joint marketing strategies, regional socioeconomic conditions, or time-varying policies. However, we clarify that the purpose of this analysis is exclusively to establish associative patterns between lottery subtypes, not to infer causality. Specifically, the pre-regression correlation tests between welfare lottery components (lotto-type Y_1^w, instant-type Y_2^w) and sports lottery components (lotto-type Y_1^s, instant-type Y_2^s) serve as a descriptive foundation for subsequent analyses, aiming to distinguish how different lottery categories co-vary in the data. Given this focus on association (rather than causal mechanisms), OLS is appropriate for capturing these data-driven relationships. We have revised the text to explicitly emphasize that all results reflect associations, not causality, aligning with the reviewer's suggestion to avoid causal implications.

2. Interpretation of Lottery Sales Co-Movement
Regarding the observed association between welfare lottery lotto-type sales and sports lottery lotto-type sales (a 1% increase in the former correlating with a 0.956% increase in the latter), we clarify that this reflects co-movement in the data rather than substitution, complementarity, or causal effects. We explicitly note that such associations may arise from shared third variables (e.g., seasonal marketing

campaigns, macroeconomic trends) but emphasize that our analysis does not disentangle these mechanisms—consistent with our focus on association. We have added a discussion acknowledging that the stability of this relationship during economic shocks remains untested, framing it as a direction for future research with causal identification methods.

3. Control Variables
As suggested, we have revised the main text to explicitly list control variables, which include per capita GDP, unemployment rate, housing prices, population size, average level of education, World Cup and COVID-19 pandemic. These are now detailed in Subsection [Econometric Model] to ensure transparency.

4. Robustness and Causal Interpretation
To reinforce the associative nature of the findings, we have clarified that the "<5% impact of sports guessing games" refers solely to data-driven correlation with lotto-type sales, not causal influence. We explicitly state that no conclusions about underlying mechanisms (e.g., consumer behavior drivers) are drawn from this association, as causal inference would require methods like IV or a panel fixed-effects model, which are beyond the scope of this descriptive analysis.

5. Limitations of Associative Analysis
We have added a discussion highlighting the limitations of relying on associative results, emphasizing that causality between lottery subtypes or with external factors (e.g., policies, economic shocks) cannot be established with the current framework. This aligns with the reviewer's advice to caution against overinterpreting correlations.

These revisions strengthen the clarity of our analytical goals, explicitly distinguish association from causality, and enhance transparency in methods and variables. Thank you for your valuable feedback, which has significantly improved the manuscript's precision and rigor.

Reviewer #2:
The author has hypothesized that high-income regions are more impacted by the online lottery sales ban (especially in sports lottery), but this opens important questions: Are these effects short-term or persistent? Does he observe substitutiontoward other gambling products (e.g., foreign online platforms or local gaming)? What are the implications for public revenue and responsible gambling policy?

Revision Response:
We appreciate Reviewer #2's thoughtful questions regarding the nature, spillover effects, and policy implications of the online lottery sales ban, which have prompted us to clarify and expand our discussion. We address these points as follows:

First, regarding the persistence of the ban’ s impact: We clarify that the online sales ban primarily reduces purchasing convenience, a barrier that may be mitigated over time by alternative channels such as expanded physical lottery outlets, phone-based sales, or promotional efforts via short-video platforms. Thus, we interpret the observed stronger impact in high-income regions as short-term—driven by immediate disruption to established online habits—rather than a persistent effect, as convenience can be partially restored through substitute channels. This nuance has been added to the discussion of results.

Second, on potential substitution toward other gambling products (e.g., foreign online platforms or local gaming): We acknowledge that investigating such spillovers would require granular data on cross-product gambling behavior, which is beyond the scope of our current dataset (focused on official lottery sales). As such, we cannot definitively confirm or rule out substitution effects and have added a note highlighting this as a limitation and valuable direction for future research with broader behavioral data.

Finally, regarding implications for public revenue and responsible gambling policy: We expand the policy discussion to emphasize that a “one-size-fits-all” ban on online sales may not be optimal. Instead, addressing root issues — such as online gambling addiction and illegal sales — through targeted measures (e.g., mandatory real-name verification, state-certified distribution channels, and stricter oversight of online platforms) could balance convenience, revenue stability, and responsible gambling. We note that while the 10-year-old ban addressed immediate concerns, advancements in digital governance (e.g., trust mechanisms similar to those in e-commerce) now offer tools to mitigate risks without eliminating online access entirely, supporting a more nuanced policy approach.

These revisions enhance the discussion of the ban’ s dynamics and policy relevance, addressing the reviewer’ s critical questions while clarifying the scope and limitations of our analysis. Thank you for highlighting these important considerations.
