# Supplementary material for: Impact of online lottery sales prohibition on the structure of lottery consumers: A time cost perspective in China
Source: PLoS One. 2025 Dec 5;20(12):e0337433. doi: 10.1371/journal.pone.0337433 (PMC12680344; doi:10.1371/journal.pone.0337433)
Supplement: Attachment — Submitted filename: Response_to_Reviewers_auresp_2.pdf [file pone.0337433.s009.pdf]

Response to Reviewers

Reviewer #2:

A brief statement regarding the different types of lottery options available in China should be mentioned in the introduction section. Sports lottery and lotto lottery are the only options? Do the consumers have the options to purchase traditional type lottery tickets? Unless a brief introduction regarding the lottery system (as is prevalent in China) is discussed it is very difficult to judge the models developed by the author. It is really needed to know, whether the consumers are required to bear other costs (like brokerage fees etc.) Are all types of lotteries are being sold online? These questions are extremely essential to provide a proper judgement regarding the assumptions, econometric works and conclusions of the manuscript. Actually a brief introduction regarding the structure of lottery system in china is needed to be discussed.

Revision Response:

We sincerely appreciate Reviewer #2's valuable feedback on supplementing the introduction of China's lottery system, which is critical for evaluating the manuscript's models, assumptions, and conclusions. To address these concerns, we have added a brief statement in the introduction section, clarifying key details of China's lottery system as follows:

China's lottery market operates under a state monopoly, exclusively administered by two entities: the China Sports Lottery (CSL) and the China Welfare Lottery (CWL). The CSL offers three main types of lottery products: numerical lotteries (e.g., Super Lotto), sports-betting games (e.g., football match result lotteries), and instant scratch cards. The CWL primarily provides traditional draw games (e.g., Double Color Ball) and instant products. In terms of distribution, online lottery sales have been banned since 2015; currently, over 95% of transactions rely on more than 200,000 physical outlets nationwide. Consumers only pay the face value of lottery tickets (e.g., ¥2 per bet for Double Color Ball) with no additional costs such as brokerage fees.

Reviewer #2:

1)The statement' Does the reduction in lottery purchasing accessibility disproportionately affect high-income versus low-income groups?' is grammatically correct but it can be improved using any of the following options

a) "Does the reduction in lottery purchasing accessibility disproportionately affect high-income or low-income groups?" or

b) "Does the reduction in lottery purchasing accessibility disproportionately impact high-income groups compared to low-income groups?"

Revision Response:

We thank Reviewer #2 for pointing out the room for improving the sentence's expression. Following the suggested option (a), we have revised the sentence in the

manuscript to: “Does the reduction in lottery purchasing accessibility disproportionately affect high-income or low-income groups?”

Reviewer #2:
‘China’s per capita expenditure ($49) exhibits marked regional heterogeneity, lagging behind high-income nations (Norway: $864; Switzerland: $632) and even trailing some middle-income economies?’ Does it mean annual consumption expenditure per capita or per capita expenditure on lottery? Clarify properly. The subsequent statement may lead one to think that it is per capita consumption on lottery, however it should to clarified properly to have an idea with respect to the later part of the statement.

Revision Response:
We appreciate Reviewer #2’s reminder to clarify the definition of “per capita expenditure,” which helps avoid ambiguity. We have revised the relevant content in the manuscript to explicitly specify the indicator, as follows:
China's annual per capita consumption expenditure on lottery products ($49) exhibits marked regional heterogeneity. This figure significantly lags behind that of high-income nations (e.g., Norway: $864; Switzerland: $632) and even some middle-income economies in terms of annual per capita lottery expenditure.
